# Management of Dermal Avulsion Injuries Using Tissue Glue in the Emergency Department: A Report of Two Cases

**DOI:** 10.7759/cureus.103032

**Published:** 2026-02-05

**Authors:** Jacqueline Yang, Chloe Ang, Hong Wei Choo, Eunizar Omar

**Affiliations:** 1 Emergency Department, Ministry of Health Holdings, Singapore, SGP; 2 Emergency Medicine, Sengkang General Hospital, Singapore, SGP

**Keywords:** dermal avulsion injury, emergency department, fingertip injury, hemostasis, tissue adhesive, tissue glue

## Abstract

Dermal avulsion injuries involve traumatic separation of the epidermal and dermal layers and commonly affect the fingertips. These injuries may present challenges in achieving hemostasis despite appearing minor.

This report describes a two-patient case series of fingertip dermal avulsion injuries managed with tissue glue in the emergency department. Two young adult males presented with persistent bleeding following household food preparation injuries that did not respond to direct pressure or adrenaline-soaked gauze. Tissue glue was applied following local hemostatic measures. Both patients achieved immediate bleeding control, experienced no major complications, and reported satisfactory cosmetic outcomes at follow-up.

In selected patients with small dermal avulsion injuries, tissue glue may offer a practical and effective option for achieving hemostasis and wound coverage in the emergency department. Larger studies are needed to better define its role and long-term outcomes.

## Introduction

Dermal avulsion injuries (DAI) result from traumatic separation of the epidermal and dermal layers and frequently involve the fingertips [1]. In the emergency department (ED), these injuries may be deceptively challenging to manage due to persistent bleeding, contamination risk, and difficulty in achieving durable wound closure. Direct pressure alone is often insufficient, prompting patients to seek emergency care despite the small size of the wound.

Avulsed skin is frequently unsuitable for reattachment due to contamination or tissue disruption, and traditional hemostatic methods may be ineffective or cumbersome. Cyanoacrylate tissue adhesives polymerize on contact with skin to form a strong protective barrier. While widely used for linear lacerations with well-approximated edges, their application in dermal avulsion injuries is less commonly described [2]. This case series describes the use of tissue glue for fingertip DAI encountered in routine ED practice. It adds to the limited literature by demonstrating a simple, reproducible technique using tissue glue to achieve rapid hemostasis and wound coverage in selected fingertip DAI in the ED.

## Case presentation

Case 1

**Figure 1:**
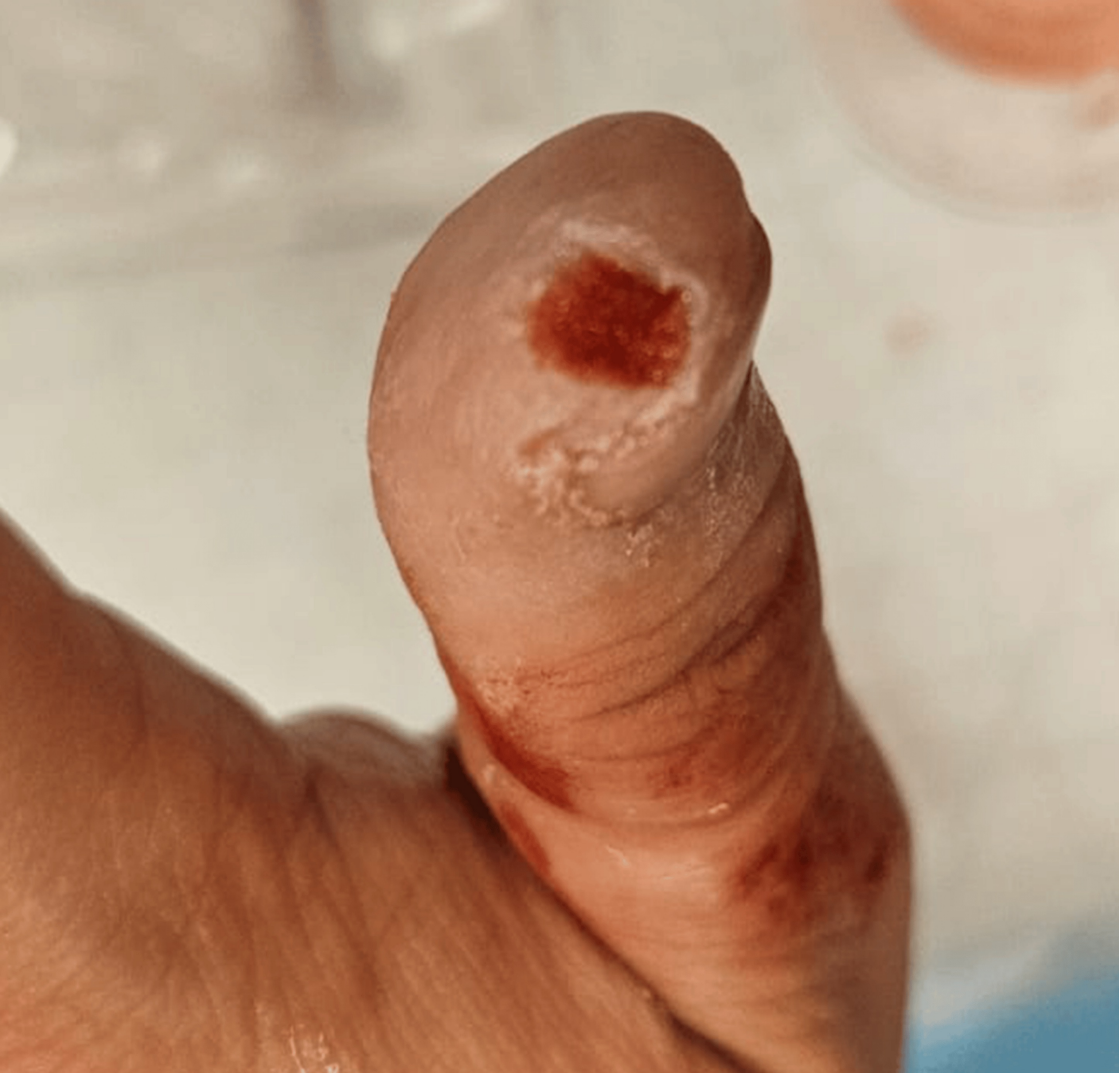
Case 1: fingertip dermal avulsion injury of the left thumb, demonstrating an ovoid defect with active bleeding

A 27-year-old Chinese male presented to the ED with active bleeding from a 1-cm ovoid dermal avulsion at the tip of his left thumb (Figure 1). The injury occurred while chopping chicken with a kitchen knife. He had no significant medical comorbidities.

**Figure 2:**
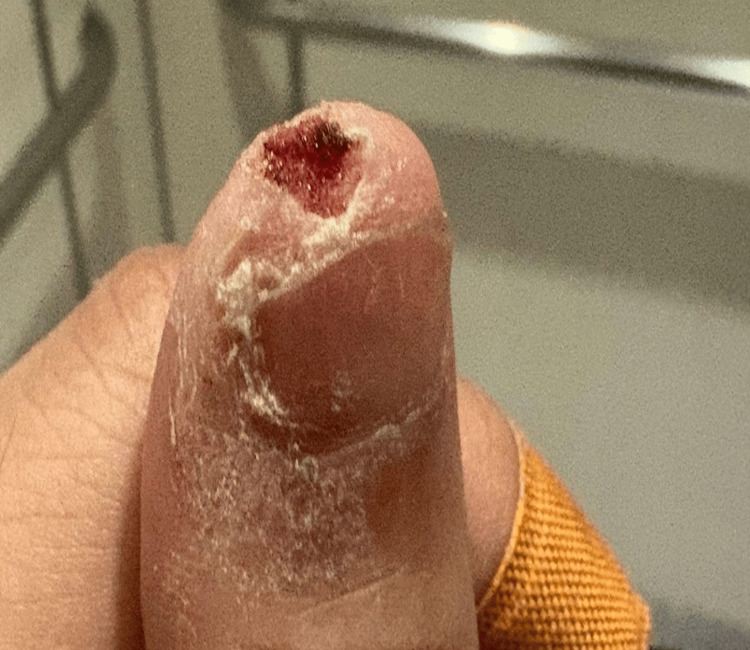
Case 1: Post tissue glue application to the left thumb dermal avulsion injury after achievement of hemostasis, illustrating coverage of the wound surface

**Figure 3:**
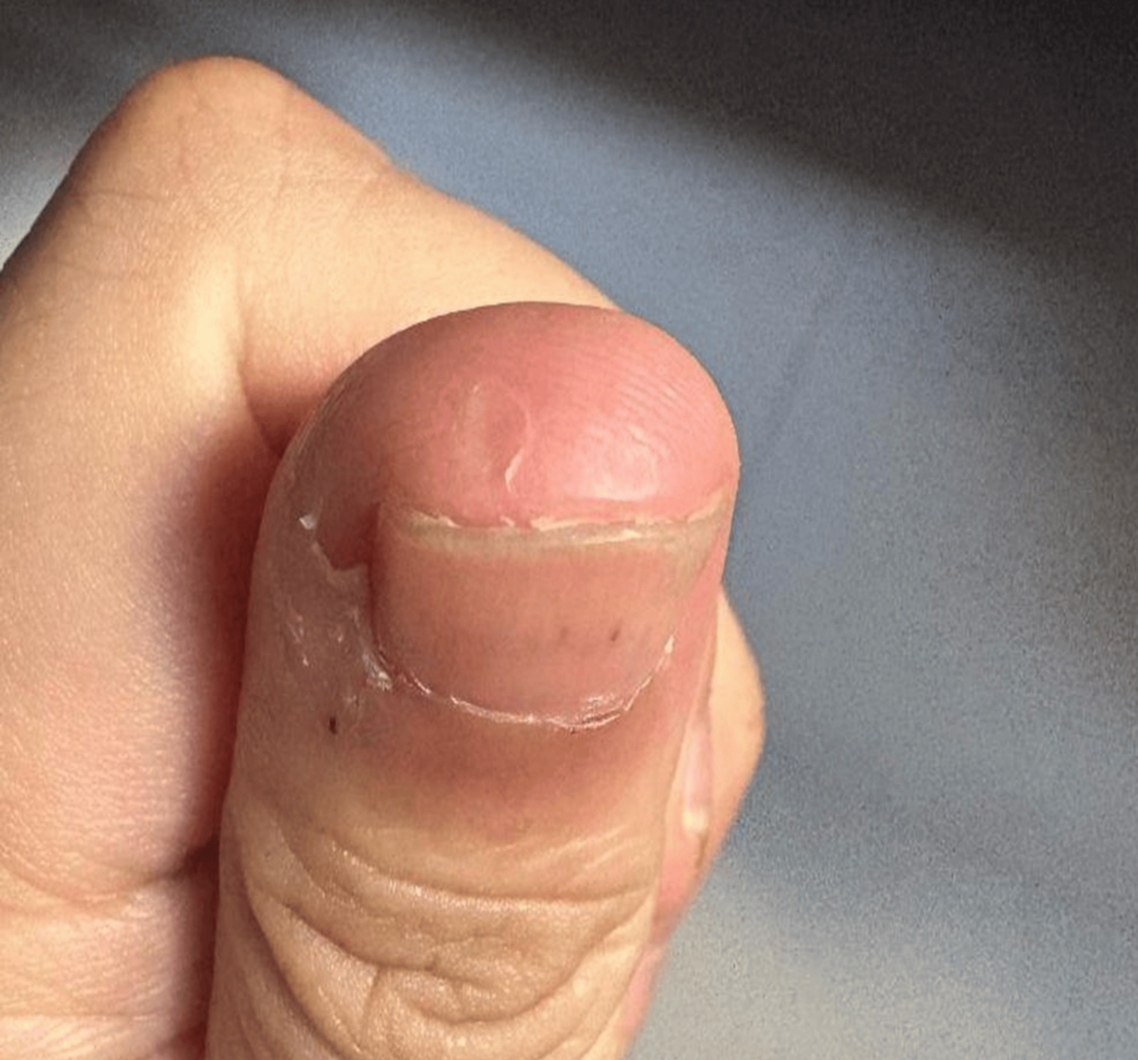
Case 1: Left thumb dermal avulsion injury at the one-month follow-up, demonstrating satisfactory wound healing without infection or rebleeding

Plain radiographs of the thumb (posteroanterior and oblique views) demonstrated no fracture, dislocation, or foreign body. Initial attempts at hemostasis using adrenaline-soaked gauze were unsuccessful. Tissue glue (Dermabond®) was subsequently applied following local hemostatic measures. Hemostasis was achieved immediately and maintained during a 30-minute observation period (Figure 2). The patient was given wound care advice and advised to return to the ED for any rebleeding or signs of infection. He did not require antibiotics or follow-up in the clinic. At one month, a follow-up call was conducted, and the patient reported no rebleeding or infection and was satisfied with the cosmetic outcome (Figure 3).

Case 2

**Figure 4:**
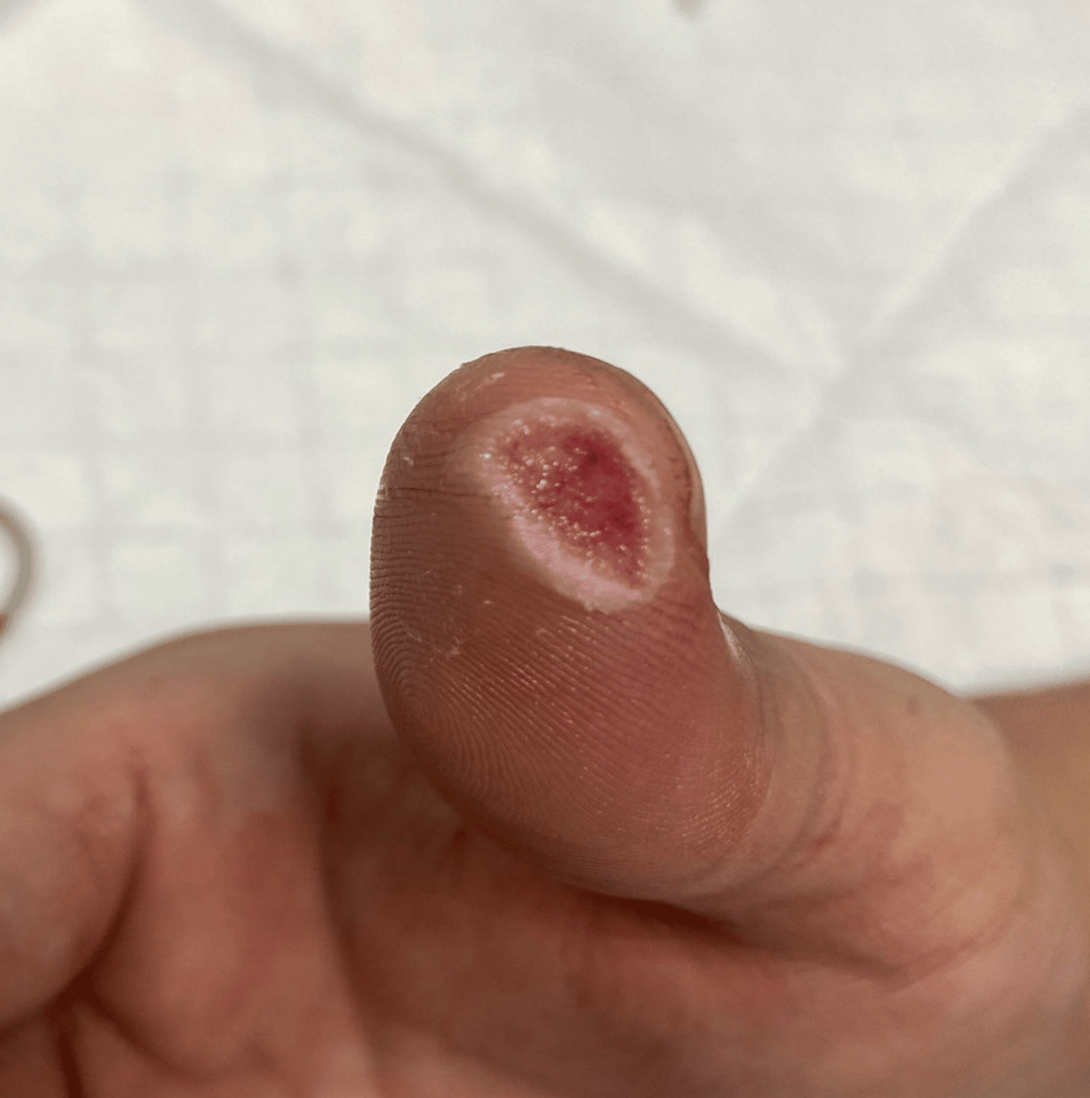
Case 2: Post tissue glue application to a dermal avulsion injury of the right thumb following persistent bleeding despite initial local measures

**Figure 5:**
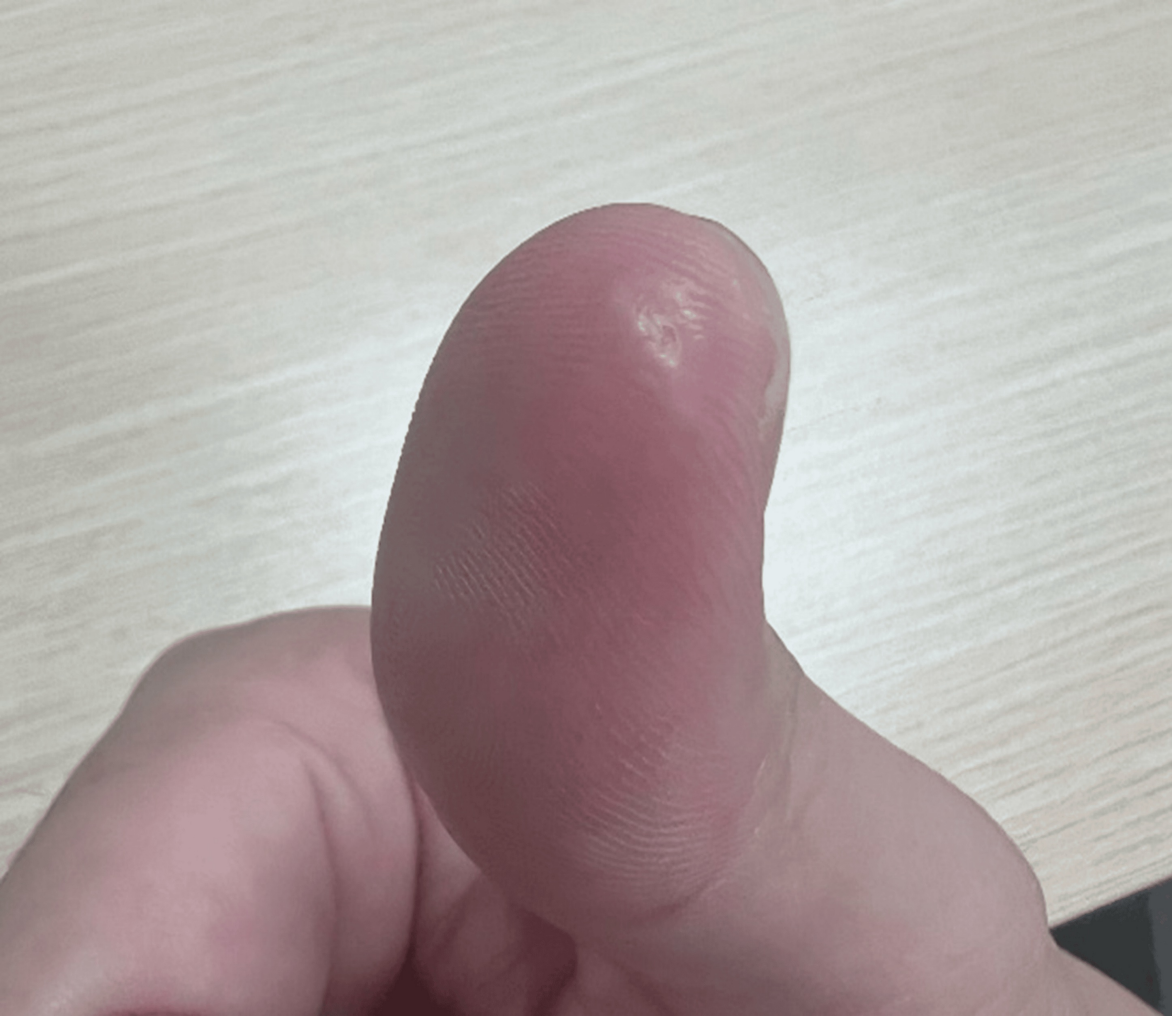
Case 2: Right thumb dermal avulsion injury at the two-month follow-up, demonstrating a healed wound with an acceptable cosmetic outcome

A 31-year-old Chinese male sustained a 0.7-cm dermal avulsion to the tip of his right thumb while using a mandolin slicer to prepare vegetables. Persistent bleeding despite an adrenaline-soaked gauze prompted the application of tissue glue (Dermabond®; J&J MedTech, New Brunswick, NJ, US) (Figure 4). The patient was also given wound care advice and advised to return to the ED for any rebleeding or signs of infection. He did not require antibiotics or follow-up in the clinic. During a two-month follow-up call, the patient reported no infection or rebleeding and noted only mild residual tingling beneath the wound. He remained satisfied with the cosmetic outcome (Figure 5).

## Discussion

Dermal avulsion injuries are common presentations in the ED and may pose management challenges when bleeding is difficult to control. Radiographic evaluation should be considered when there is concern for fracture or retained foreign bodies, and adequate wound irrigation is essential to reduce infection risk [3].

It is important to distinguish DAI from other digital injuries such as tip amputation and pulp lacerations. Fingertip DAI differ fundamentally from both fingertip amputations and pulp lacerations in terms of tissue loss, anatomical involvement, and management. Dermal avulsions involve partial loss of epidermis and dermis due to shearing forces or incising injury, with the preservation of deeper structures such as the distal phalanx and nail bed. DAI typically heals by secondary intention without the need for formal closure. In contrast, fingertip amputations represent true loss of tissue, often including the bone and nail bed, and frequently require surgical reconstruction or revision. Pulp lacerations, while sometimes deep and painful, are linear or stellate cuts in which tissue remains viable and is amenable to primary closure. Distinguishing between these entities is important, as DAI are commonly managed conservatively, whereas amputations and pulp lacerations often necessitate operative or sutured repair [4].

**Figure 6:**
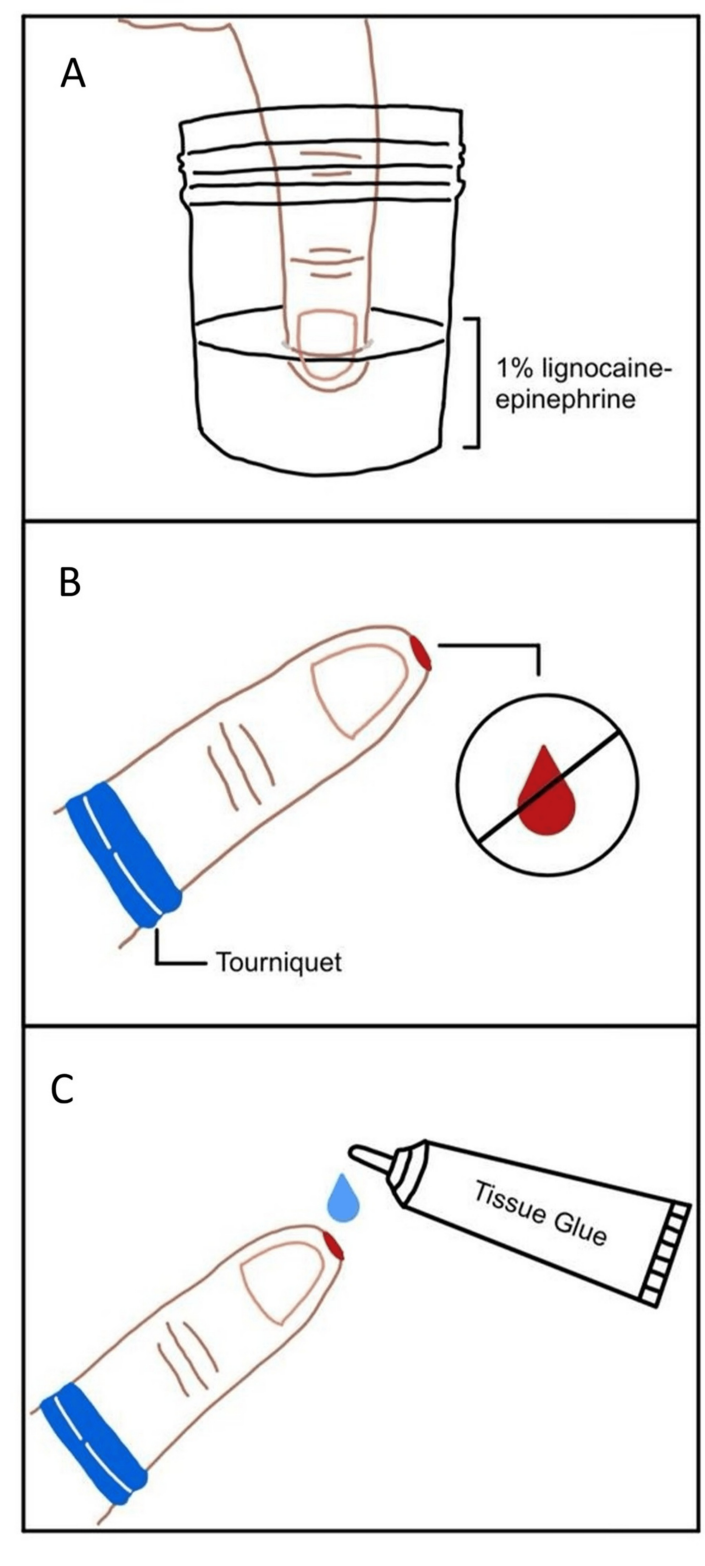
Stepwise technique for achieving hemostasis and applying tissue glue in fingertip dermal avulsion injuries (A) The fingertip is first soaked in 10-20mL 1% lignocaine with epinephrine to aid vasoconstriction and analgesia. (B) A proximal digital tourniquet is then applied, and the limb is elevated and exsanguinated to create a bloodless field. (C) Tissue glue is subsequently applied in thin layers to the wound surface, extending 1 mm around the wound edge after hemostasis is achieved. Image Credits: Jacqueline Yang

Tissue glue repair of DAI has been previously described, albeit with varying levels of technical detail. Prior to tissue glue application to the wound, local hemostatic measures are employed. A bloodless field is created by soaking the affected fingertip in 10-20 mL of 1% lignocaine with adrenaline for approximately five minutes, followed by an elevation of the digit and application of a proximal digital tourniquet [1]. Where required, gentle circumferential compression of the digit is performed to facilitate exsanguination. A small container, such as a medicine cup or specimen bottle, may be used to contain the solution. Tourniquet time is kept within safe limits, generally not exceeding 90 minutes for fingers or toes [5]. Tissue glue is then applied in thin, sequential layers, allowing each layer to fully dry and polymerize before tourniquet release [2]. The number of layers depends on wound depth, although approximately 10-15 layers are typically required until the defect is adequately sealed. Care is taken to extend the glue coverage approximately 1 mm beyond the wound margins to ensure an adequate circumferential seal. To accelerate polymerization, a nasal cannula connected to air or oxygen may be used to gently blow over the wound surface. This approach facilitates rapid bleeding control and avoids the need for suturing or prolonged healing by secondary intention. Figure 6 outlines the step-by-step technique.

Once the tissue adhesive has dried completely, the tourniquet is removed, and the wound is inspected for residual bleeding. If required, the tourniquet may be reapplied and additional adhesive applied to achieve an adequate seal. After complete drying, a dry, waterproof dressing may be applied, ensuring that no adherent surface contacts the repaired area. Patients should be advised to keep the wound clean and dry, avoid rubbing the site, and refrain from exposure to soaps or chemicals to maintain the integrity of the adhesive barrier. If rebleeding or infection occurs, the patient should be advised to see a doctor for wound review. Mild residual surface contour deformity and disruption of the fingerprint pattern may be observed and reflect the initial injury rather than the repair technique; patients should be counselled accordingly.

This case series illustrates the practical application of tissue glue specifically for small DAI encountered in routine ED practice. Although traditionally recommended for clean, linear wounds with well-approximated edges, limited evidence and clinical experience suggest that tissue glue may be used in selected small dermal avulsion injuries without exposed bone or tendon [6]. Furthermore, cyanoacrylate tissue adhesives exhibit modest intrinsic antimicrobial activity through the release of small amounts of formaldehyde and cyanoacetate during polymerization, and by forming an occlusive barrier that limits bacterial ingress, with infection rates comparable to sutured closure in clean wounds [7].

While tissue glue offers rapid, non-invasive hemostasis, potential risks should be considered. Inadequate wound cleansing prior to application may theoretically trap surface contamination, underscoring the importance of meticulous irrigation and inspection before glue deployment. Occlusive sealing may theoretically delay epithelialization in some cases; however, in this series, wound healing was achieved within a clinically acceptable timeframe without infection [8]. Formal comparison of healing rates was beyond the scope of this report, and further comparative studies are required to elucidate potential differences in time to epithelialization. The mild residual tingling reported in Case 2 is a recognized symptom following superficial fingertip trauma and may reflect local nerve irritation from the initial injury rather than a direct effect of tissue adhesive.

Patient selection considerations

Based on the authors’ experience and existing literature, tissue glue may be considered in selected DAI characterized by small defect size (generally <1 cm), clean wound edges, and absence of exposed bone, tendon, or fracture. Adequate hemostasis must be achievable prior to application, and wounds should not be grossly contaminated [6]. This approach is most suitable for low-tension areas, such as the fingertip pulp, and should be avoided in high-mobility regions, animal bites, or wounds with significant contamination. Tissue glue should be avoided in contaminated wounds, animal bites, mucosal surfaces, or high-mobility areas, such as joints, due to the risk of infection or wound dehiscence [2].

Comparison with alternative approaches

Management options for fingertip DAI include healing by secondary intention, suturing, or the use of hemostatic dressings. Suturing small avulsion defects may be technically challenging due to limited tissue for edge approximation and may increase procedure time and patient discomfort. Healing by secondary intention often necessitates prolonged wound care and may be associated with ongoing bleeding or delayed epithelialization. Hemostatic dressings, while effective, may incur higher direct costs to patients and often require repeat applications and follow-up visits. In selected cases, tissue adhesive offers a minimally invasive alternative that provides immediate hemostasis and wound coverage without the need for sutures, frequent dressing changes, or routine outpatient follow-up, and may represent a more convenient and comfortable option for patients [9].

Limitations and future directions

This report comprises two illustrative case reports and was not methodologically designed or powered for outcome comparison or effectiveness evaluation. Its purpose is descriptive and pictorial, intended to highlight the feasibility and practical application of an underused hemostatic technique rather than to draw comparative or causal inferences. Objective outcome measures were not prospectively collected, and follow-up relied on patient-reported outcomes. Future studies with prospective design and comparator groups are needed to formally evaluate clinical outcomes and define the role of tissue glue in fingertip DAI.

## Conclusions

In this two-patient case series, we illustrated an underused technique for the management of fingertip dermal avulsion injuries. Tissue glue application enabled rapid hemostasis and acceptable cosmetic outcomes in these cases, without observed short-term complications. This approach may be considered a practical option for carefully selected patients in the ED. Larger prospective studies are required to better define its role, healing trajectory, and longer-term outcomes.
